# Administrative Data Linkage to Evaluate a Quality Improvement Program in Acute Stroke Care, Georgia, 2006–2009

**DOI:** 10.5888/pcd12.140238

**Published:** 2015-01-15

**Authors:** Moges Seyoum Ido, Rana Bayakly, Michael Frankel, Rodney Lyn, Ike S. Okosun

**Affiliations:** Author Affiliations: Rana Bayakly, Georgia Department of Public Health, Atlanta, Georgia; Michael Frankel, Emory University, Atlanta, Georgia; Rodney Lyn, Ike S. Okosun, Georgia State University, Atlanta, Georgia.

## Abstract

**Introduction:**

Tracking the vital status of stroke patients through death data is one approach to assessing the impact of quality improvement in stroke care. We assessed the feasibility of linking Georgia hospital discharge data with mortality data to evaluate the effect of participation in the Georgia Coverdell Acute Stroke Registry on survival rates among acute ischemic stroke patients.

**Methods:**

Multistage probabilistic matching, using a fine-grained record integration and linkage software program and combinations of key variables, was used to link Georgia hospital discharge data for 2005 through 2009 with mortality data for 2006 through 2010. Data from patients admitted with principal diagnoses of acute ischemic stroke were analyzed by using the extended Cox proportional hazard model. The survival times of patients cared for by hospitals participating in the stroke registry and of those treated at nonparticipating hospitals were compared.

**Results:**

Average age of the 50,579 patients analyzed was 69 years, and 56% of patients were treated in Georgia Coverdell Acute Stroke Registry hospitals. Thirty-day and 365-day mortality after first admission for stroke were 8.1% and 18.5%, respectively. Patients treated at nonparticipating facilities had a hazard ratio for death of 1.14 (95% confidence interval, 1.03–1.26; *P* = .01) after the first week of admission compared with patients cared for by hospitals participating in the registry.

**Conclusion:**

Hospital discharge data can be linked with death data to assess the impact of clinical-level or community-level chronic disease control initiatives. Hospitals need to undertake quality improvement activities for a better patient outcome.

## Introduction

Assessing the impact of chronic disease programs and the quality of clinical care for patients with chronic diseases is essential to identify areas for improvement in care and to demonstrate the level and nature of improvements already made (1). The American Heart Association/American College of Cardiology Working Group on Quality of Care and Outcomes Research in Cardiovascular Disease and Stroke advocates measuring the short-term and long-term outcomes of quality of care for stroke patients as a way of determining the impact of related chronic disease programs (2). Tracking the vital status of patients with chronic disease, who may be seen at different health facilities, by using death data is a promising method for assessing the overall quality of care for chronic diseases (1).

Administrative data such as hospital discharge data and death data are great resources for public health studies (3–5). These are population-based databases that can be used to assess the quality of stroke care because they include all population groups. Administrative data are easy to access, and they provide longitudinal information for passive follow-up and trend analyses.

The Georgia Coverdell Acute Stroke Registry (GCASR) is a part of a national stroke registry program, the Paul Coverdell National Acute Stroke Registry. The national registry has the long-term goal of reducing premature deaths attributable to stroke and preventing stroke disability and recurrent stroke through ensuring the highest quality of acute stroke care to all Americans. GCASR was launched by the Georgia Department of Public Health in 2005 in partnership with other stakeholders. We sought to assess the feasibility of linking mortality data from the Georgia Department of Public Health Office of Vital Records with hospital discharge data from the Georgia Hospital Association’s Georgia Discharge Data System (GDDS) and to evaluate the impact of participation in a state-based registry program on survival of patients with acute ischemic stroke.

## Methods

### Georgia death records and Georgia hospital discharge data

The Georgia Department of Public Health Office of Vital Records is responsible for collecting information about deaths among Georgians by using the death certificates. The death certificate contains information on individuals’ demographic characteristics, residence, underlying possible causes of death, location of death, and death date. Each year, more than 67,000 Georgians die, and 98% of the deaths occur within the state of Georgia.

The GDDS is housed at the Georgia Hospital Association and has information on all inpatients discharged from nonfederal short-stay hospitals in Georgia. GDDS gathers more than a million records per year. GDDS and mortality data share common variables including age, sex, race, residence information, and a quasi-unique subject identifier (LONGID) that facilitates the data linkage.

The feasibility of data linkage is based on the assumption that the variable LONGID was sufficiently specific to distinguish each subject in the data sources. The LONGID is a 15-digit alphanumeric unique code created from letters of patients’ first and last names, birth date, and sex. We tested accuracy of data linkage by using data from GDDS for 1,494 Georgia patients who were admitted to a hospital for acute stroke and who died as a result (*International Classification of Diseases, Ninth Revision* [ICD-9]) codes 430–436) in 2006 and for 3,598 patients with similar age characteristics (patients with malignant neoplasm of respiratory and intrathoracic organs: ICD-9 codes 160–165) but who were alive in 2006. Patients with similar age characteristics were chosen because personal name patterns in a given community may change through time.

The test data set was then linked with the Georgia mortality data for 2006 by using a multistage deterministic and probabilistic matching algorithm and various combinations of key variables (Table 1 and Table 2). We used fine-grained record integration and linkage software for matching, and we excluded duplicate entries using the LONGID, admission and discharge dates, and facility codes (6). Degrees of linkage between hospital discharge data and mortality data were determined (Table 3).

**Table 1:** Algorithm for Merging the Georgia Discharge Data System With the Georgia Mortality Data of the Same Calendar Year

Linkage Step	Linking Variable	Distance Metric (Approve/Disapprove Level)	Condition Weight,^a^ %	Acceptance Level,^b^ %
Step I	LONGID^c^	Edit distance (0.05/0.15^d^)	70	80
	Residence county	Equal fields Boolean distance	15	
	Race	Equal fields Boolean distance	10	
	Sex	Equal fields Boolean distance	5	

Step II	Name^e^	Edit distance (0.15/0.3)	40	80
	Birth date	Date distance (±0 d)	30	
	Discharge date	Date distance (±0 d)	20	
	Residence zip code^f^	Equal fields Boolean distance	10	

Step III	Name^e^	Edit distance (0.15/0.3)	40	95
	Age, y	Numeric distance (±0)	10	
	Discharge date	Date distance (±0 d)	20	
	Residence zip code^f^	Equal fields Boolean distance	15	
	Race	Equal fields Boolean distance	10	
	Sex	Equal fields Boolean distance	5	

Step IV	Birth date	Date distance (±0 d)	35	100
	Discharge date	Date distance (±0 d)	25	
	Residence county	Equal fields Boolean distance	25	
	Race	Equal fields Boolean distance	10	
	Sex	Equal fields Boolean distance	5	

a Proportional weight for each element in the linkage step.

b Total match score at which records are considered to be linked.

c 15-digit alphanumeric code created from letters of patients’ first and last names, birth date, and sex.

d The proportion of mismatched characters used to determine whether the records are considered to be linked.

e Refers to a 6-digit code derived from names.

f 5-digit zip code.

**Table 2:** Algorithm for Merging the Georgia Discharge Data System With the Georgia Mortality Data From Different Calendar Years

Linkage Step	Linking Variable	Distance Metric (Approve/Disapprove Level)	Condition Weight,^a^ %	Acceptance Level,^b^ %
Step I	LONGID^c^	Edit distance (0.05/0.15)	70	80
	Residence county	Equal fields Boolean distance	15	
	Race	Equal fields Boolean distance	10	
	Sex	Equal fields Boolean distance	5	

Step II	Name^d^	Equal fields Boolean distance	40	81
	Birth date	Date distance (±0 d)	30	
	Residence zip code^e^	Equal fields Boolean distance	15	
	Race	Equal fields Boolean distance	10	
	Sex	Equal fields Boolean distance	5	

Step III	Name^d^	Edit distance (0.15/0.3)	40	100
	Age	Numeric distance (±0)	30	
	Residence county	Equal fields Boolean distance	15	
	Race	Equal fields Boolean distance	10	
	Sex	Equal fields Boolean distance	5	

a Proportional weight for each element in the linkage step.

b Total of condition weights at which records are considered to be linked.

c 15-digit alphanumeric code created from letters of patients’ first and last names, birth date, and sex.

d Refers to a 6-digit code derived from names.

e 5-digit zip code.

**Table 3:** Agreement in the Matching Variables of the Linked Georgia Hospital Discharge Data and Georgia Mortality Data

Variable	Agreement, %	
	Test Data and 2006 Death Data	2006–2009 Hospital Discharge and 2006–2010 Death Data
LONGID^a^	85.8	91.3
Birth date	94.5	96.2
Name^b^	91.9	98.3
Sex	99.2	99.8
Age	98.1	— d
Race	95.2	96.8
Residence county	91.0	88.3
Residence zip code^c^	62.0	62.6
Facility	93.6	— d
Discharge date or date of death	92.6	— d

a 15-digit alphanumeric code created from letters of patients’ first and last names, birth date, and sex.

b Refers to a 6-digit code derived from names.

c 5-digit zip code.

d Not all records are expected to match.

### Assessment of impact of stroke registry — survival analysis

We used the 2005 through 2009 GDDS and the 2006 through 2010 Georgia Office of Vital Records mortality data to examine the survival rates of acute ischemic stroke patients. Patients admitted to nonfederal acute care and critical access facilities with the principal diagnosis ICD-9 codes 433 and 434 were identified and linked to the death data. Death and survival time from the index admission date, regardless of the underlying cause of death, were the outcome variables. We believe that care in the first few hours after stroke symptom onset determines the stroke patient’s subsequent health condition, so we attributed the outcome to the facility of first stroke admission. Patients were labeled to have had a first stroke admission in 2006 if they were not admitted for any type of stroke, ICD-9 codes 430–438, in 2005.

We defined enrollment in GCASR if the hospital actively participated in data entry and quality improvement activities. We considered patients to have had stroke care by a GCASR facility if the hospital in which patients were admitted was enrolled in the registry. Patients who were treated at any time before a facility was enrolled or after it withdrew its participation were counted as patients treated by a non-GCASR hospital. We included patient’s sociodemographic characteristics such as age, sex, race, insurance status, and length of hospital stay, and hospital features including number of beds and location as covariates in the analysis. On the basis of the number of beds, we classified hospitals as small (<100 beds), medium-small (100–249 beds), medium-large (250–399 beds) and large hospitals (≥400 beds). We used the Rural-Urban Commuting Area classification of location to classify hospitals geographically into metropolitan (codes 1–3) and nonmetropolitan (codes >3) (7).

Comorbidities were included in the analyses to adjust for disease severity. We used the Healthcare Cost and Utilization Project software from the Agency for Healthcare Research and Quality (US Department of Health and Human Services) to define comorbidities for each patient based on the ICD-9 codes in the hospital discharge data (8,9). Patients’ readmission status before either the end of the follow-up period or the patient’s death was captured from the hospital discharge data, and we classified patients as either not having been readmitted, readmitted to the same hospital, or readmitted to a different hospital. If patients were admitted to a different hospital within a day after their index or first admission date we considered their status as a transfer rather than a readmission, and they were excluded from the analysis. All the variables used in the analyses refer to what was documented at the first stroke admission except for the date of death. To have stable estimates, we excluded stroke patients from hospitals with fewer than 15 patients over the study period.

### Statistical analysis

We analyzed the data by using SAS for Windows (version 9.3, SAS Institute, Inc). We assessed the sensitivity of the linkage procedure based on the proportion of stroke-related in-hospital deaths that were captured by the 2006 Georgia vital records mortality data. We determined specificity by the proportion of subjects who were admitted in 2007 having a malignant neoplasm of the respiratory organs that were linked to any of the records in the 2006 death file. We assessed patient and hospital characteristics descriptively and tested differences between patients treated at GCASR participating and nonparticipating hospitals using χ^2^ tests for nominal variables and Wilcoxon tests for quantitative variables.

We assessed the proportional hazard assumption graphically and through the goodness-of-fit test for correlation between the Schoenfield residuals and failure time (10). We repeated the graphic assessment using the log–negative log of survival curves after adjusting for covariates. The GCASR participation variable did not satisfy the proportional hazard assumption. Thus, we analyzed survival time in correlated data using the extended Cox proportional hazard model with the robust sandwich estimate option to estimate the marginal covariate effects. We performed the analysis with and without censoring at 1 year. Results are presented indicating the hazard ratio for death in the first year after the seventh day of the first stroke admission date by different patient and hospital characteristics, including participation in GCASR.

## Results

### Data linkage test for accuracy

Of the 1,494 acute stroke patients with an in-hospital death recorded in the 2006 hospital discharge data, 1,381 (92.4%) were identified in the 2006 death data, whereas none of the 3,598 patients with malignant neoplasm of respiratory and intra-thoracic organs diagnosed in 2007 were linked to the 2006 death data. Agreements between hospital discharge records and death data were high (>91%) for demographic variables, facility (93.6%), and discharge or death dates (92.6%) (Table 3).

### Impact of participation in state-based stroke registry: survival analysis

From the initial 50,937 patients listed, 358 were excluded because 269 were considered transfers and 89 were from hospitals with fewer than 15 cases. Analysis was performed for 50,579 acute ischemic stroke patients (Table 4) admitted to 131 acute care and critical access hospitals in Georgia to assess the impact of participation in GCASR during 2006 to 2009. Most (52%) were women, and whites accounted for two-thirds (66%) of the patients. The mean age for first stroke admission was 69 years. Most (64%) had Medicare as their principal health insurance coverage. The median hospital length of stay was 3 days.

**Table 4:** Characteristics of Acute Ischemic Stroke Patients (n = 50,579) Cared for by Georgia Coverdell Acute Stroke Registry Participating and Nonparticipating Hospitals, Georgia Hospital Discharge Data, 2006–2009, and Georgia Mortality Data, 2006–2010

Characteristics	Treatment Location			*P* Value^b^
	All Hospitals	GCASR Hospitals	Non-GCASR Hospitals^a^	
**Age, y, mean (SD)**	68.7 (13.9)	68.2 (13.9)	69.3 (13.9)	.12
**Sex, n (%)**				
Male	24,494 (48.4)	13,948 (49.7)	10,546 (46.9)	<.001
Female	26,085 (51.6)	14,129 (50.3)	11,956 (53.1)	
**Race, n (%)**				
White	33,619 (66.5)	18,813 (67.0)	14,806 (65.8)	.63
Black	15,695 (31.0)	8,445 (30.1)	7,250 (32.2)	
Other	1,265 (2.5)	819 (2.9)	446 (2.0)	
**Primary insurance coverage, n (%)**				
Medicare	32,438 (64.1)	17,531 (62.4)	14,907 (66.3)	.31
Medicaid	2,877 (5.7)	1,687 (6.0)	1,190 (5.3)	
Private	10,329 (20.4)	6,088 (21.7)	4,241 (18.8)	
Self-pay	3,607 (7.1)	2,097 (7.5)	1,510 (6.7)	
All others	1,328 (2.6)	674 (2.4)	654 (2.9)	
**Length of stay, d, median (interquartile range)**	3.0 (2–6)	2.8 (1.3–5.4)	3.2 (1.7–5.6)	.80
**Hospital size, n (%)**				
<100 beds	70 (53.4)	22 (36.7)	48 (67.6)	<.001
100–249 beds	29 (22.1)	11 (18.3)	18 (25.4)	
250–399 beds	15 (11.5)	12 (20.0)	3 (4.2)	
≥400 beds	17 (13.0)	15 (25.0)	2 (2.8)	
**Hospital location, n (%)^c^**				
Metropolitan	62 (47.3)	40 (66.7)	22 (31.0)	<.001
Nonmetropolitan	69 (52.7)	20 (33.3)	49 (69.0)	
**Calendar year, n (%)**				
2006	12,331 (24.4)	4,743 (16.9)	7,588 (33.7)	<.001
2007	12,959 (25.6)	7,175 (25.5)	5,784 (25.7)	
2008	12,849 (25.4)	7,972 (28.4)	4,877 (21.7)	
2009	12,440 (24.6)	8,187 (29.2)	4,253 (18.9)	
**No. (%) of deaths**				
Discharge	1,940 (3.8)	1,000 (3.6)	940 (4.2)	.08
30 days	4,114 (8.1)	2,105 (7.5)	2,009 (8.9)	<.001
365 days	9,350 (18.5)	4,740 (16.9)	4,610 (20.5)	<.001
End of follow-up	14,699 (29.1)	7,281 (25.9)	7,418 (33.0)	<.001

Abbreviation: GCASR, Georgia Coverdell Acute Stroke Registry; SD, standard deviation.

a Non-GCASR hospitals are those that never participated in GCASR from 2006 through 2009.

b χ^2^ and Wilcoxon tests were applied for nominal and quantitative variables, respectively.

c Based on Rural-Urban Commuting Area classification of location to classify hospitals geographically as metropolitan (codes 1–3) or nonmetropolitan (codes >3) (7).

GCASR-participating hospitals treated 56% of the ischemic stroke patients (n = 28,077), and there were no statistical differences in age, hospital length of stay, proportion of various racial groups, or proportion of subjects with insurance coverage between patients treated at GCASR and non-GCASR hospitals (Table 4). However, non-GCASR hospitals were more likely to see female stroke patients, have less than 100 beds, to be in nonmetropolitan areas, and record more stroke-related deaths at 30 and 365 days following stroke admissions. The overall mortality at 30 days and 365 days after the first admission were 8.1% and 18.5%, respectively.

The extended Cox model indicated that patients treated at non-GCASR hospitals had a hazard ratio of 1.14 (95% confidence interval [CI], 1.03–1.26) from the eighth day after admission to 1 year after admission (Table 5). A similar hazard ratio (1.13; 95% CI, 1.04–1.22) was observed when no cutoff date was applied. Similarly, older patients and those treated in nonmetropolitan hospitals had a higher hazard ratio than their counterparts. Patients with a private insurance or self-pay had a lower hazard ratio than did Medicare patients. In addition, hospitals with fewer than 100 beds and longer hospital stays for patients were independently associated with subsequent death (Table 5).

**Table 5:** Relative Risk for Death for Georgians With Acute Ischemic Stroke, Georgia Hospital Discharge Data, 2006–2009, and Georgia Mortality Data, 2006–2010

Characteristic	Hazard Ratio^a^ in the First Year Post Stroke Admission	
	Estimate (95% CI)	*P* Value^b^
**Location of treatment**		
Hospital participating in GCASR	1 [Reference]	
Hospital not participating in GCASR	1.14 (1.03–1.26)	.01
**Sex**		
Female	1 [Reference]	
Male	0.93 (0.89–0.98)	.004
**Age group, y**		
<45	1 [Reference]	
45–64	1.34 (1.14–1.57)	<.001
65–79	2.18 (1.83–2.62)	<.001
≥80	5.45 (4.53–6.56)	<.001
**Race**		
White	1 [Reference]	
Other	1.03 (0.96–1.11)	.36
**Primary insurance coverage**		
Medicare	1 [Reference]	
Medicaid	1.06 (0.94–1.19)	.35
Private	0.75 (0.67–0.84)	<.001
Self-pay	0.62 (0.51–0.75)	<.001
All others	0.91 (0.80–1.19)	.84
**Length of stay, d**	1.017 (1.013–1.022)	<.001
**Hospital size, n (%)**		
≥400 beds	1 [Reference]	
250–399 beds	1.05 (0.91–1.21)	.48
100–249 beds	1.04 (0.92–1.18)	.54
<100 beds	1.17 (1.02–1.33)	.02
**Hospital location^c^**		
Metropolitan	1 [Reference]	
Nonmetropolitan	1.11 (1.03–1.21)	.009
**Calendar year**		
2009	1 [Reference]	
2008	1.02 (0.95–1.09)	.64
2007	1.09 (1.02–1.17)	.007
2006	1.09 (1.02–1.18)	.02

Abbreviation: CI, confidence interval; GCASR, Georgia Coverdell Acute Stroke Registry.

a Adjusted for comorbidities.

b χ^2^ and Wilcoxon tests were applied for nominal and quantitative variables, respectively.

c Based on Rural-Urban Commuting Area classification of location to classify hospitals geographically as metropolitan (codes 1–3) or nonmetropolitan (codes >3) (7).

## Discussion

Acute ischemic stroke patients cared for by hospitals participating in GCASR had a better outcome than their counterparts in nonparticipating hospitals. This study found a modest (14%) increase in the hazard ratio for death in the first year for patients treated at non-GCASR participating facilities. Several studies have shown that quality improvement efforts result in improved stroke patient care (11–14). This study, however, demonstrated that a state-based initiative based on the collaborative effort of professionals who are willing to share their expertise and exchange best practices results in tangible benefit to the community served.

Patients treated at non-GCASR facilities continued to have the same hazard ratio throughout their follow-up time, indicating perhaps that the clinical care provided to patients at their first stroke episode influenced their risk of mortality in the subsequent years. Regardless of whether hospitals participated in the GCASR, patient outcomes throughout Georgia improved with time. Compared with patients who had an acute ischemic stroke in 2009, patients during 2006 through 2007 had a 9% higher risk of dying during the first year after the index admission. Development of new treatment guidelines and their implementation by health care providers may have contributed to the reduction in mortality; however, it is impossible to rule out a possible spillover effect of the GCASR initiatives to nonparticipating facilities.

There was no meaningful difference in outcomes among hospitals of different size except for small hospitals (<100 beds) where patients had a 17% higher risk of mortality. Hospitals participating in GCASR tended to be metropolitan and larger, and although our analyses adjusted for these 2 variables, differences attributable to other variables between the 2 hospital groups cannot be ruled out. It is not possible, thus, to associate the reduction in hazard ratio among the GCASR hospitals entirely to the quality improvement initiatives undertaken by the registry. In future studies, linking the registry data (where interventions received by patients are documented) to the hospital discharge and death data will be helpful to associate the clinical care information with patient outcome.

The yield from the linkage procedure was sufficient to assess the impact of the quality improvement program. There would be patients who died but were not picked by the matching procedure; however, failure to link was not related to the type of hospital where patients were treated in the test data set. Failure to link gives a lower estimate of the actual mortality but does not introduce bias in the study’s effect measure. Studies elsewhere reported different rates of mortality for ischemic stroke (15–19). The mortality at 1 month poststroke admission ranges from 9% in Australian and Israeli studies to 17% in a Rochester, Minnesota, study. Also, the 1-year mortality has been reported to vary from less than 10% in Japanese and Taiwanese studies to 29% in the Minnesota study. The observed differences may be due to variations in study methodology, population characteristics, and quality of patient care. The 1-year mortality estimate observed in this analysis (18.5%) lies between extreme values that have been reported by other investigators, thus indicating that the linkage procedure was sufficiently sensitive and may even be a reasonable approach to estimate mortality and survival rates across the course of stroke patient care. We believe our estimates may be lower than expected rates because the data linkage may not have captured all patients who died in the given period, particularly those who died outside the state of Georgia.

This study has limitations, some of which are inherent to any method that assesses the effect of a quality improvement intervention. It is difficult to define the time when the effect of such an intervention wanes, and several factors contribute to the overall well-being of a patient through time. Survival of acute ischemic stroke patients depends on factors such as patient and hospital characteristics, the time from symptom onset to arrival at the hospital, disease severity, the quality of service received from the health care facility on first encounter, the quality of rehabilitation services, and the quality of life once the patient is discharged from a hospital. This analysis took into account most of the prehospital discharge factors except for time elapsed between symptom onset and arrival at the hospital. In addition, we did not have information on postdischarge rehabilitation and quality of life.

Although administrative data may lack consistent case definitions from one data set to another and the use of ICD-9 codes may not capture all possible acute stroke patients, the effect of misclassification is minimal in studies addressing the impact of hospitals’ participation in a quality improvement registry, because misclassifications are more likely to be nondifferential and would only reduce the effect measure toward the null value. Moreover, this study may not have completely captured disease severity, which is the main predictor of mortality. Different indices, including the National Institute of Health Stroke Scale, have been suggested by researchers to predict mortality, but there is no consensus index (20–24). Each one has its own merit in terms of feasibility of data collection, availability for data collection, and discriminatory power of fatal outcome. Several studies used the comorbidity measure, initially developed by Elixhauser et al (8) in various disease conditions (25–30), and Zhu and Hill have demonstrated its usefulness in stroke as well (31). It is, thus, reasonable and practical to use comorbidity measures to account for disease severity.

State-based hospital discharge data and death data can be linked and are excellent for estimating survival or risk for mortality, outcome measures that are helpful to assess the impact of clinical-level or community-level chronic disease control initiatives. The results of this study show that participation in a state-based stroke registry for improving the quality of care is associated with reduced mortality from acute ischemic stroke. Thus, hospitals should be encouraged either to participate in a structured program of quality improvement such as state-based registries or undertake their own quality improvement to provide the best possible evidence-based care to their patients for a better outcome.
